# Optimization of spicy red pepper paste (*Awaze*) formulation by D‐optimal mixture design

**DOI:** 10.1002/fsn3.3874

**Published:** 2023-12-07

**Authors:** Biadge Kefale, Mulugeta Admasu Delele, Solomon Workneh Fanta, Solomon Abate

**Affiliations:** ^1^ Bahir Dar Institute of Technology, Faculty of Chemical and Food Engineering Bahir Dar University Bahir Dar Ethiopia; ^2^ Ethiopian Institute of Agricultural Research, Holeta Agricultural Research Centre Food Science and Nutrition Research Holeta Ethiopia; ^3^ Ethiopian Institute of Agricultural Research, Head Quarter, Food Science and Nutrition Research Addis Ababa Ethiopia

**Keywords:** *awaze*, D‐optimal, nutritional content, optimization, spice

## Abstract

The aim of this study was to produce spicy red pepper paste (*Awaze*) by addition of various sources of antioxidant, mineral, and fiber and optimize better processing methods of the paste. For this purpose, D‐optimal mixture design was used, *Awaze* considering color value (a*), antioxidant content, mineral content, fiber content, chewiness, and viscosity of the paste as the main parameters. Various properties of the optimized formulation were evaluated. The optimal formulation contained 65.66% red pepper, 10% garlic, 19.08% red onion, and 5.25% ginger. In the optimized formulation, the redness value (a*) increased by more than 3.12 times compared to the control with the average antioxidant activity (44.6%). The optimal formulation had a higher fiber content, chewiness, and viscosity value compared to control which is probably related to the ingredient proportion difference. Due to the higher nutritional and processing quality obtained, this formulation can be suggested for commercial and household producers as a guide to manufacture *Awaze*. The results obtained indicate that it is possible to production of *Awaze* with high nutritional value and improved processing quality by utilizing a blend of red pepper, garlic, red onion, and ginger. Therefore, this formulation stands as a viable recommendation for both commercial enterprises and household producers due to its demonstrated ability to yield Awaze with enhanced nutritional content and superior processing quality.

## Graphical abstract

Novelty: To our knowledge, this is the first report showing spicy red pepper paste (Awaze) formulated and optimized using red pepper, garlic, red onion, and ginger as main ingredients, and by selected parameters for Awaze quality. Following the method of D‐optimal mixture design technique a highly acceptable Awaze in terms of physicochemical, mineral content, and texture profile was obtained, that will be used as a guide to manufacture Awaze for household or commercial users.

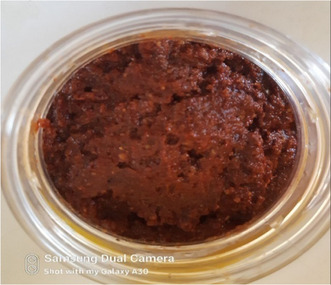

## 1 INTRODUCTION

*Awaze* is a fermented red pepper paste, and is one of the most important spice‐based traditional Ethiopian foods, especially in the northern part of the country. It is generally used as a sauce for stew (*wot*) making, with boiled potato, with roasted meat, and served with *Injera* during coffee ceremony and as seasoning in spicy foods. Spices are important ingredients of functional foods owing to their beneficial properties for human health. Their benefits are mainly related to the presence of bioactive compounds, antioxidant, and anti‐inflammatory activities. In Ethiopia, spices are commonly used in cuisine and medicine. The spices that are frequently used as main ingredients for the preparation of traditional spicy red pepper paste *(Awaze)* are red pepper, garlic, red onion, and ginger (Kefale et al., 2023). These spices exert biological properties that are linked to maintaining good health and treating diseases (Hewlings & Kalman, 2017).

Red pepper and red pepper‐based products possess a high nutritional value, due to its content of different types of bioactive compounds such as antioxidants, minerals, fiber and vitamin C, specific aroma, flavor, and color (Zhuang et al., 2012). Dried and milled red peppers are used in meat, bakery products, instant soup, herb mixes, seasonings, and sauces in the food industry (Tekin & Baslar, 2018). Garlic is rich source of potassium, phosphorous, magnesium, sodium, calcium, iron, and selenium (Haciseferoǧullari et al., 2005). Garlic also contains vitamins especially thiamin with high bioavailability owing to some specific sulfur containing components. The nutritional, medicinal, and sensory quality (taste) of onion makes it a very popular vegetable all over the world. It has high content of flavonoids, macro nutrients, and micronutrients (Lanzotti, 2006).

Red onion is a good source of fiber, crude protein, minerals, and antioxidants. Chemical analysis of onion showed high amount of oil (20.4%), fiber (22.4%), crude protein (24.8%), calcium (175/100 mg), and potassium (1010/100 mg) (Dini et al., 2008). Ginger has health‐related properties that include antioxidant, anti‐inflammatory, antimicrobial, anticancer, cardiovascular protective, respiratory protective, anti‐obesity, and anti‐diabetic (Mao et al., 2019). Nutritional, sensorial quality, and functional characteristics are important parameters to produce functional foods that possess beneficial effects for health and are accepted by consumers (Aggarwal et al., 2016).

In Ethiopia there are several traditionally prepared spice‐based foods such as red pepper paste (*Awaze)*, stew*(shero) wot*, *Mekelesha*, *Datta*, *Selejo*, *and Bekolt*. Spices used for the preparation of these spice‐based foods are red onion, ginger, garlic, cardamom, fenugreek, clove, cinnamon, kemune, long pepper, coriander, thyme, nutmeg, basil, white cumin, black cumin, white cumin, rue, rosemary, ground pepper, black mustard, safflowers, bean flour, potted water, and salt (Demeke et al., 2020).

Previous studies on laboratory‐made and commercial *Awaze* quality characteristics reported, particularly, on the microbial quality and some physicochemical quality of *Awaze*. The fermentation and microbial quality of *Awaze* was reported by Idris et al., 2001. The behavior of E.coli O157:H7 during fermentation of *Awaze* at different storage temperatures was studied by Tsegaye et al., 2004.Probiotics property consisting of acid and bile tolerance, antibacterial activity, and antimicrobial susceptibility of lactic acid bacteria isolated from *Awaze*, the probiotics property and antibiotic resistance of lactic acid bacteria isolated from *Awaze* evaluated in vitro were reported by Tigu et al., 2016 and Dessalegn & Ashenafi, 2010.

Mixture design has been applied as an important method for the development and optimization of foods. When several food ingredients are mixed, it is difficult to analyze foods by other available methods, determining their mixing ratio can be difficult. In a food product with several mixed ingredients, a mixture experiment design is used to identify ingredients that affect the product quality and to determine the optimal mixing ratio that maximize or minimize the response level of the ingredients (Jang et al., 2011). An optimal mixture design is proved as an efficient tool to determine the best ingredient combination in food formulation. This method is widely used to find the optimal formulation in various food products by considering the best food characteristics (Al Hagbani & Nazzal, 2018).

Many Ethiopian women at household level use various proportions of red pepper, garlic, red onion, and ginger during the preparation of *Awaze*. There is lack of scientific information about the optimum proportion of the main ingredients that are used for the preparation of *Awaze* at household and commercial level.

Therefore, this study was conducted to evaluate the a) possibility of producing a new functional *Awaze* by increased antioxidant, ash, and fiber content and b) the physicochemical properties, nutritional content, and texture profile of enriched *Awaze*. To the best of our Knowledge, this is the first study in the literature focused on the production of *Awaze* designed for consumers by the combination of red pepper, garlic, red onion, and ginger.

## 2 MATERIALS AND METHODS

### 2.1 Materials

Dried red pepper sample was collected from Bure district in September, 2022, Amhara region, Ethiopia, the place is known for its high volume production of red pepper as described in Dessie et al., 2019. Matured red onion, ginger, and garlic were obtained from a vegetable improvement center, Holeta Agricultural research center. The other ingredients (spices), cardamom, fenugreek, clove, cinnamon, *kemune*, long pepper, *gewze*, coriander, thyme, basil, white cumin, black cumin, rue, rosemary, and ground pepper were purchased from Holeta spice market in September, 2022, Oromia region, Ethiopia. The collected samples were transported to Holeta Agricultural research center, Food science and Nutrition research laboratory for *Awaze* paste product development. Analytical grade chemicals and distilled water have been used in various steps of *Awaze* paste preparation and physicochemical quality analysis.

### 2.2 Experimental design

Maximum and minimum levels of independent variables were first investigated by doing a preliminary survey at various household‐made *Awaze* paste producers in northwest Amhara, areas where the product is frequently produced and consumed. Based on the survey of the raw materials (ingredients) that are used in the preparation of *Awaze* paste, the ingredients that were used in a higher proportion were red pepper, red onion, garlic, and ginger. It was found that a maximum of 30% garlic, 20% red onion, and 10% ginger will be substituted with red pepper. It was found that substituting more than 30% garlic and 10% ginger to make *Awaze* paste results in a color change, whereas mixing more than 20% red onion decreases the shelf life of *Awaze* paste.

Accordingly, a total of 15 formulations were generated using D‐optimal mixture design with four ingredients (red pepper, garlic, red onion, and ginger) using Design‐Expert 13.0.1(Stat‐Ease Inc.) software which was used to define the optimum proportion of the enriched *Awaze* paste formulation. In this study, D‐optimal design was used with four components: red pepper, garlic, red onion, and ginger. Table 1 displays the composition of each blend calculated from the experimental design. The amount of the components was selected based on preliminary survey result of household producers of *Awaze* paste (red pepper: 60%–90%, garlic: 10%–30%, red onion: 5%–20%, and ginger: 5%–10%). Design‐Expert software designed 15 samples (Table 1). Effect of red pepper, garlic, red onion, and ginger on the attribute of *Awaze* paste was investigated and the optimum mixture was selected. Formulated *Awaze* paste and control sample were compared based on selected parameters for *Awaze* paste, including color (a* value), viscosity, antioxidant activity, total ash content, fiber, and chewiness of the product (Tables 1 and 2).

**TABLE 1:** Proportions of ingredients used for the optimization of *Awaze* paste formulation (100%).

Code	Parameters	Low level (%)	High level (%)
A	Percentage of red pepper	60	90
B	Percentage of garlic	10	30
C	Percentage of red onion	5	20
D	Percentage of ginger	5	10

**TABLE 2:** Experimental designs for red pepper, garlic, red onion, and ginger composite *Awaze* paste using D‐optimal mixture design.

Formulation	Mixture components (%)			
	Red pepper	Garlic	Red onion	Ginger
1	60	20.22	12.01	7.77
2	67.28	19.37	8.28	5.07
3	70.13	10.00	9.87	10
4	68.84	13.99	12.16	5.01
5	60	30.00	5.00	5
6	76.39	10.00	8.60	5.01
7	64.55	15.46	10.17	9.82
8	60	15.00	20.00	5
9	68.88	16.10	5.02	10
10	65.27	10.00	17.70	7.03
11	74.39	13.33	5.00	7.28
12	63.27	24.68	7.03	5.02
13	60	10.13	19.87	10
14	64.15	20.85	5.00	10
15	60	14.81	15.20	9.99

#### 2.2.1 Control (household made) sample

At household‐level preparation (unknown formulation), the ingredients used are the same for all districts in the region for *Awaze* paste preparation but the ratio of the ingredient used is different. In order to optimize the difference in the mixing ratio, the experiment is conducted, and the area which is popular for the utilization of *Awaze* paste is selected and used as a control sample to compare to the current experiment. Household‐made *Awaze* paste (unknown formulation) collected from Bure district, northwest Amhara, areas where the product is frequently produced and consumed or utilized, was used as a control sample.

#### 2.2.2 Optimization and contour plots

The optimal ingredient mixing ratio was designated and the experimental points predicted by the numerical optimization and graphical optimization of ingredients in the mixture. For simultaneous optimization each response must have a low and high value assigned to each goal. On the work sheet, the ‘goal’ field for response must be one of 5 choices: ‘None’, ‘maximum’, ‘minimum’, ‘target’, or ‘in range. Factors will always be included in the optimization. For graphical optimization, the minimum and maximum of each response were determined and contour plots were super‐imposed within the possible ranges, after which the best area was selected.

### 2.3 Preparation of *Awaze* paste

#### 2.3.1 Drying, roasting, and milling of ingredient

The preparation of *Awaze* paste was preformed following the traditional household‐made method as described in (Idris et al., 2001). The ingredients collected for *Awaze* paste preparation were dry spices and wet spices. Spices are classified as dry spices, such as red pepper, cardamom, fenugreek, clove, cinnamon, *kemune*, long pepper, coriander, white cumin, black cumin, and ground pepper, and as wet spices such as red onion, garlic, ginger, thyme, basil, rue, and rosemary. The processing of *Awaze* paste ingredients was preformed following the traditional methods described below. The dried red pepper was gently pulverized with a wooden mortar and pestle. Fresh un‐dried garlic, red onion, and ginger were peeled and washed. A small proportion of other weight spices, such as thyme, basil, rue, rosemary, was powdered separately using a wooden mortar and pestle. The powdered wet spices were sun dried by thinly spreading on a clean surface.

**FIGURE 1:**
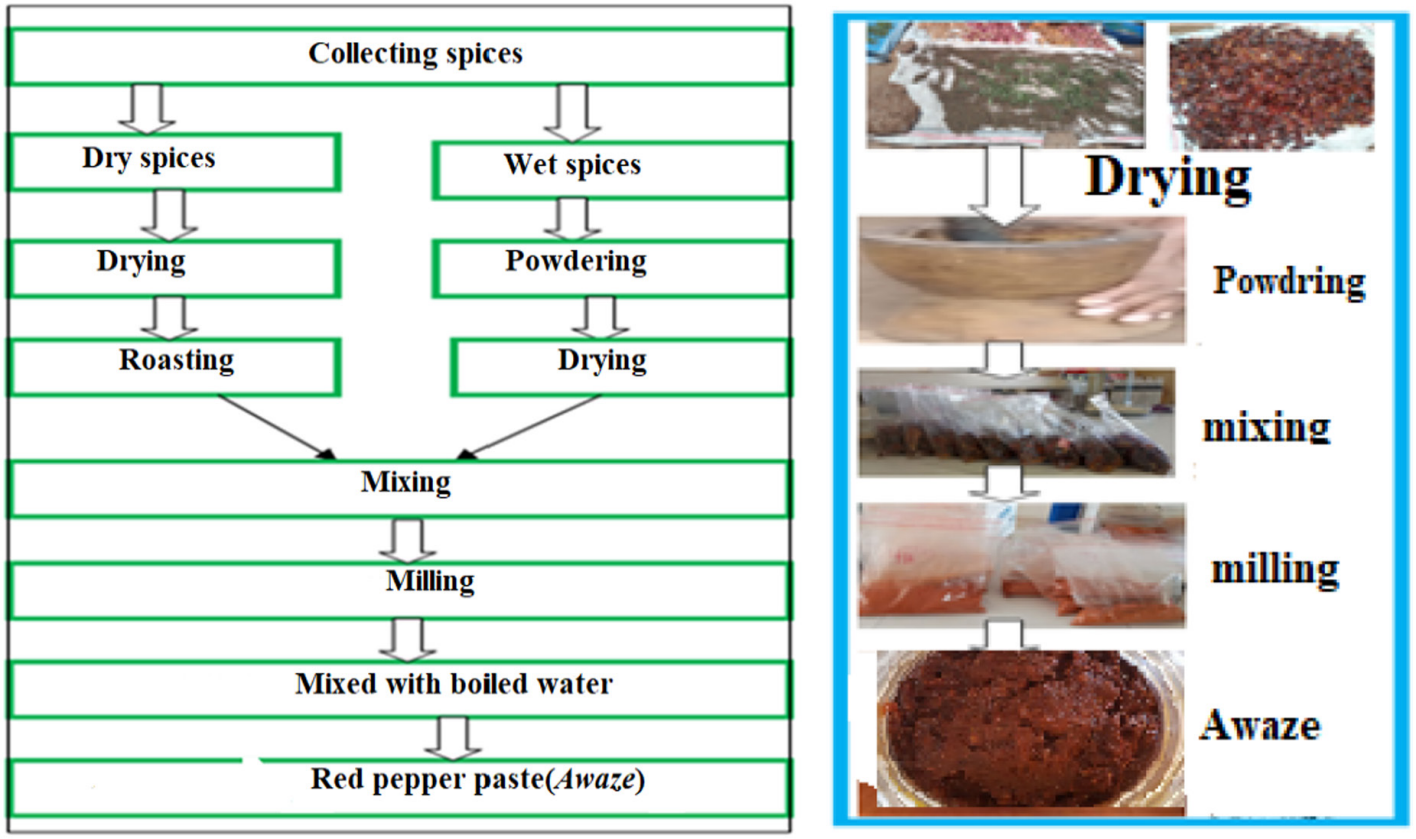
Processing steps for the preparation of *Awaze*.

Dry spices were lightly roasted and mixed with dried wet spices following the formula. The composite blends (independent variables) that contained red pepper, garlic, red onion, and ginger were prepared using the formula shown in Table 2. Controlled variables that include 5 g cardamom, 5 g fenugreek, 2.5 g white cumin, 2.5 g basil, 1.25 g black cumin, 1.25 g *mekelesha*, 1 g rue, 0.5 g coriander, 0.4 g rosemary, 0.4 g thyme, 20 g salt were added constantly for each formulation. The mixed spices were milled using laboratory hammer mill (Perten Instruments, Finland). Then, *Awaze* paste was prepared by mixing 100 g composite powder with 150 mL of boiled water in a 400 mL capacity screw–cap bottle until it acquired a thick consistency as shown in (Figure 1). Control sample (household made) was collected from Bure district, Amhara region, areas where the product is frequently produced and consumed to compare with the formulated *Awaze* paste.

### 2.4 Evaluation of physicochemical property of *Awaze* paste

Physicochemical property color (L*, a*, b*) chroma, hue angle, ash content, fiber content, viscosity, and antioxidant activity of *Awaze* paste were evaluated according to official methods.

#### 2.4.1 Color value (L*,a*,b*, chroma, and hue angle)

The L*, a*, b* color values of the formulated paste (*Awaze*) were observed by Aeros Hunter colorimeter following the method described in (Li et al., 2016). The Aeros Hunter colorimeter was calibrated using the manufacturer's standard white plate. The color scale used was L*, a*, b*, chroma(C*), and hue angle(H*). The coordinate L* measures values between white (100) and black (0). The redness (a*) color value measured values between red (+127) and green (−128), while b* measures between yellow (+127) and blue (−128). Chroma (C*) and hue angle (H*) were estimated from L*, a*, and b* values by using equations described in (Pathare et al., 2013).

(1) $$C^{*}=\sqrt{a{*}^{2}+b{*}^{2}}$$

(2) $$h^{*}=\tan^{-1}\left(\frac{b^{*}}{a^{*}}\right)$$

where, C* = chroma, h* = hue angle, a* = redness, b* = yellowness.

#### 2.4.2 Total ash and crude fiber content

Total ash content and crude fiber were determined according to the association of official analytical chemists method (AOAC, 1990) no. 923.03 and 962.09, respectively.

#### 2.4.3 Viscosity

A 3 g *Awaze* paste sample was dissolved in 5 mL distilled water, and the viscosity of the solution was determined using Brookfield (model Lv‐3, Middleboro, MA 02346, USA) set at 60 rpm and a temperature of 25°C (Rahman, 1986).

#### 2.4.4 DPPH radical scavenging activity

Methanolic extracts were prepared as reported previously by (Luthria et al., 2006). 400 mg of *Awaze* paste sample was placed in a 15 mL centrifuge tube with 5 mL of the solvent mixture MeOH: H_2_O (80:20, % v/v). The vials were then placed in a sonicator bath at 25°C temperature for 30 min. The mixture was centrifuged and the supernatant was transferred into a 10 mL volumetric flask. The residue was re‐suspended in 5 mL of MeOH:H_2_O (80:20, %v/v), gently mixed manually and sonicated for an additional 30 min followed by centrifugation. The supernatant was combined with the initial extract and the volume of combined supernatant was made up to 10 mL with the extraction solvent and appropriate aliquots of extracts were filtered and assayed for antioxidant activity. Antioxidant activity was evaluated by measuring the radical scavenging effect of spicy red pepper paste (*Awaze*) sample methanolic extracts toward the 2,2‐diphenyl‐1‐picrylhydracyl (DPPH) as reported previously by (Sharifi et al., 2015). Five mL of a 0.1 mM methanol solution of DPPH was added to 20 μL of methanol extracts of *Awaze* paste samples. The tubes were allowed to stand at 27°C for 20 min. The decrease in absorbance at 517 nm was recorded in a spectrophotometer (Shimadzu UV–vis spectrophotometer 1900). Radical scavenging activity was expressed as inhibition percentage and was calculated using the following formula:

(3) $$\left(\%\right)\;\text{Radical scavenging activity}=\left(\frac{\text{Absorbance control}–\text{Absorbance sample}}{\text{Absorbance control}}\right)\;x\;100$$

#### 2.4.5 Mineral content

The mineral contents (Fe, Zn, K, Ca, and Mg) of the formulated sample and control sample were determined by atomic absorption spectrophotometer (Model: Agilent serious, America) by using the method (AOAC, 1990). A sample (0.5 g) was digested by using a muffle furnace at 550 degree centigrade for 5 hours until the residue ash became white. The ash sample was digested by using concentrated HCl. The digested sample was diluted up to 100 mL for mineral analysis.

(4) $$\text{Mineral content}\left(\mathrm{ppm}\right)=\frac{\left(\text{conc}.\mathrm{AAS}-\text{blank}\right)\times \mathrm{TV}\times \mathrm{df}}{\text{weight of sample}}$$

where TV=Volume marked by 100 mL volumetric flask, df = dilution factor.

#### 2.4.6 Texture profile analysis (TPA)

The texture profile of *Awaze* paste was measured using a Texture Analyzer (model: TA. XT. Plus) (Stable Micro Systems, UK), based on the method described in Chen and Rosenthal (2015)) using P‐2 (cylindrical probe having 2 mm diameter) with pre‐test speed, and the test speeds were stated as 2,1, 2 mm/sec, respectively. Compression was a distance mode of 5 mm and load cell was 50 kg. Two compressions in a raw were applied on the samples. The test produced the hardness, adhesiveness, cohesiveness, springiness, gumminess, and chewiness of *Awaze* paste was measured. The texture profile of *Awaze* paste was determined using the following approaches:

Hardness (N) is the maximum height of the first peak on the first compression.

Adhesiveness (N.s) describes the work for overcoming the force of attraction between the area of samples coming in contact with each other.

Cohesiveness is the ratio of the second compression to the first compression positive area

(5) $$\text{Cohesiveness}=\frac{A2}{A1}$$

Springiness is measured by dividing the distance of the detected height of the product on the second compression by the original compression distance.

Gumminess (N) is the product of hardness and cohesiveness (strength required to chew dawn).

Chewiness (N) is the product of gumminess and springiness (measure of the energy spent in the chew down process) as per the procedure of (Cardoso et al., 2009)**.**

### 2.5 Data analysis

All the formulation values were presented as the mean ± standard deviation. Statistical analysis was performed using SPSS software (IBM, Chicago, IL, USA). One way analysis of variance and Duncan multiple range test (IBM SPSS Version 24) were used to explore the significant difference between the formulations with 95% confidence interval at (*p* < .05). Correlation coefficients were analyzed using Minitab software (Version 17). Numerical optimization was employed using statistical software Design‐Expert 13.0® from Stat‐Ease Inc. with criteria of maximum red onion while red pepper, garlic, and ginger were kept in range. The contour plot for the selected variables was constructed to determine the best formulations for the chosen response.

## 3 RESULT AND DISCUSSION

### 3.1 Effect of red pepper, garlic, red onion, and ginger proportion on color value of *Awaze* paste

The surface color of *Awaze* was measured using a colorimeter and is presented in Table 4. There is a significant difference at *p* < .05 in L*, a*, b*, chroma, and hue angle for the formulated paste samples. The color values of lightness (L*), redness (a*), and yellowness (b*) of the products and control sample were in the range of 5.3–19.9, 5.85–23.4, and 4.15–28.1, respectively. Color is an essential food quality for consumer acceptability and it is highly dependent on the ingredient types used. The degree of redness (a*) was found to have a positive value ranging from 5.85 to 23.4. The difference in color value of the formulated paste could be attributed to the differences in mixing ratio of the powders. The degree of color value difference in the paste sample is affected by the type of processing, packaging material, and storage temperature (Mo et al., 2015). Following an analysis of the 15 experimental results, the redness value (a*) reached a maximal value of 23.4 for a mixing ratio of red pepper, garlic, red onion, and ginger of 74.39%, 13.3%, 5.0%, and 7.28%, respectively, whereas the control sample (household made) had the lowest value (5.85). The results revealed that the combination of red pepper, garlic, red onion, and ginger increased the redness (a*) value of *Awaze* paste by up to 4 times compared to the control sample. The low value of the control sample could be due to the long storage period of the product. A previous study on fermented red pepper‐based products revealed that the color value is associated with the microbial composition, which metabolizes the complex and converts it into simple molecules responsible for a unique taste, aroma, and color (Yang et al., 2018). The color values of *Awaze* paste that was obtained in this study were in agreement with the study that was conducted on another red pepper‐based paste product (*gochujang*) (Ramalingam., 2022). Similar redness (a*) value for Korean red pepper paste was reported (15.13–31.34) (Oh et al., 2011). Similarly, previous study reported L* value (28.77–34.93), a* value (2.58–11.03), and b* value (4.09–7.74) of Korean fermented red pepper paste during storage (Park et al., 2010). Generally a higher value of a* indicates the degree of redness color of *Awaze* paste which is a criteria for best quality product.

**FIGURE 2:**
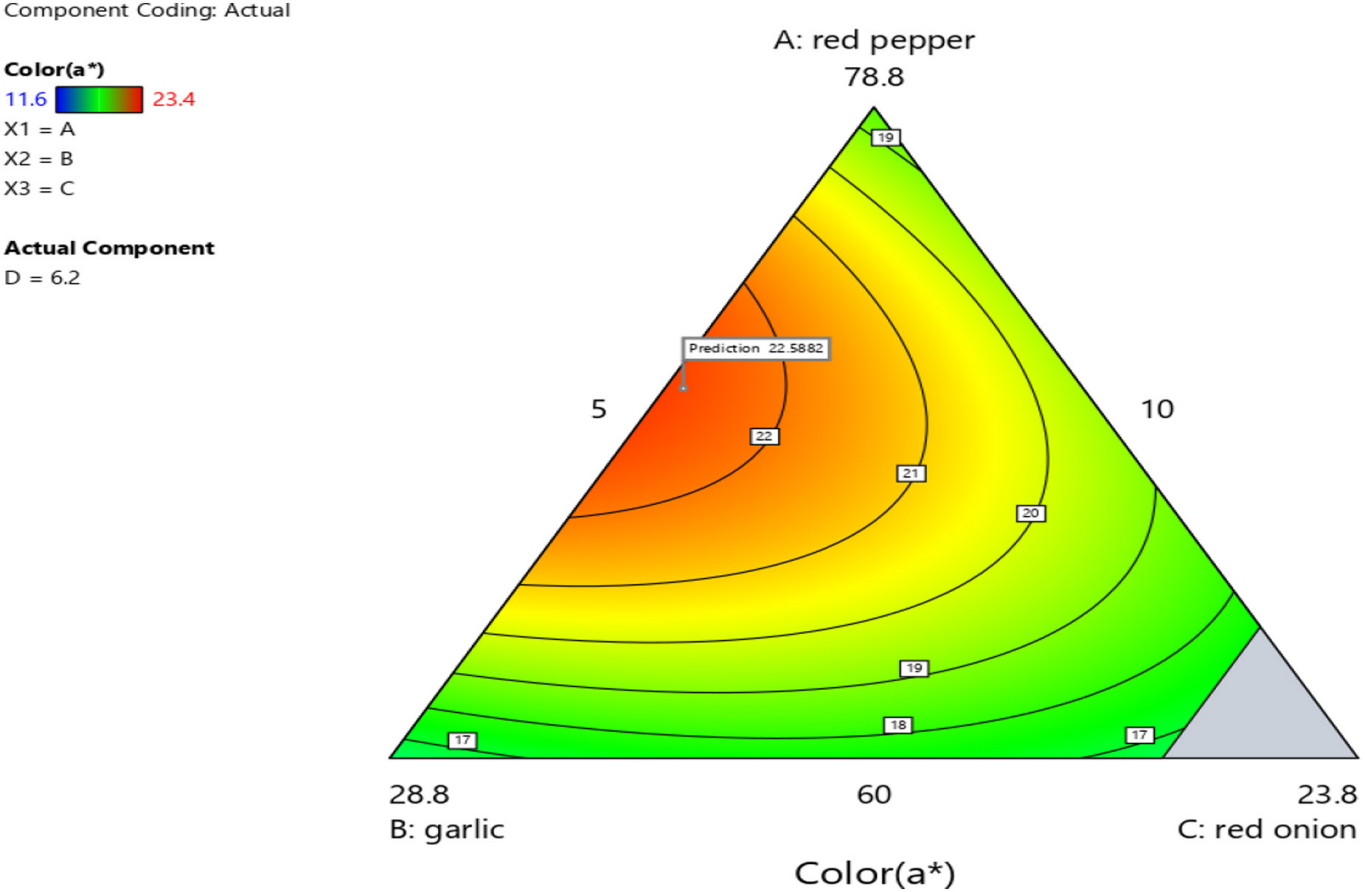
The effect of blending ratio on color value (a*) of spicy red pepper paste (*Awaze)* obtained using actual components as shown by contour plot.

The contour plot In Figure 2 indicated that with increasing the mixing ratio of red pepper and red onion, the degree of redness was significantly increased. In contrast, an increase in garlic and ginger proportion decreased the color value of *Awaze* paste. The results showed the redness of *Awaze* paste increases with an increase in mixing ratio of red pepper and red onion.

### 3.2 Effect of red pepper, garlic, red onion, and ginger proportion on ash content of *Awaze* paste

**FIGURE 3:**
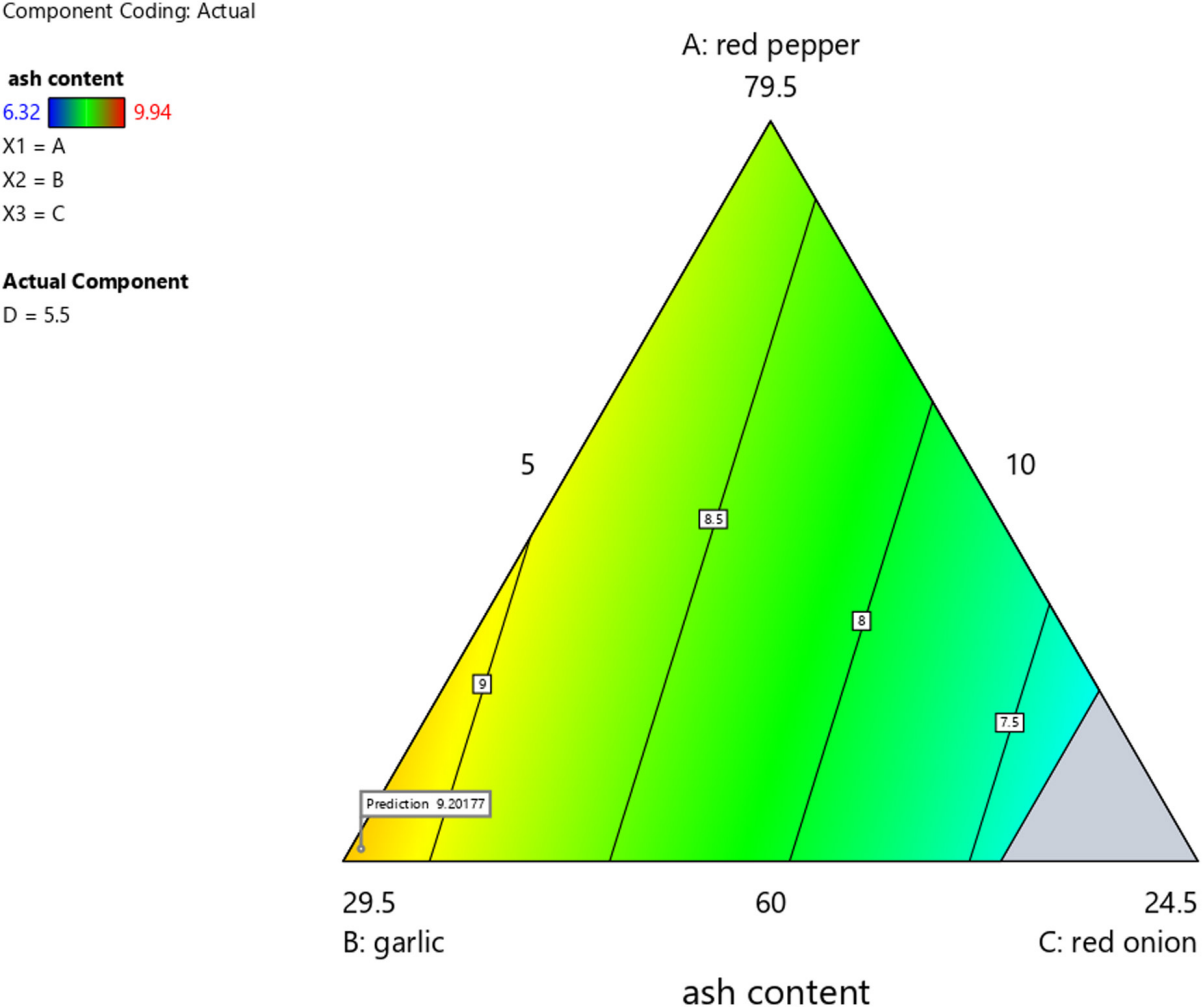
The effect of blending ratio on ash content of spicy red pepper paste (*Awaze)* obtained using actual components as shown by contour plots.

As the ash content analysis showed (Table 5), red pepper, garlic, red onion, and ginger had a strong effect on the ash content of *Awaze* paste. The ash content of *Awaze* paste samples showed significant difference for the formulated paste samples (*p* < .05) (Table 5).The ash content gives an indication of the mineral composition of food materials. In this study, the ash values of the formulated *Awaze* paste and control sample ranged 6.32%–9.94%. At a mixing ratio of 68.8% red pepper, 16.1% garlic, 5.02% red onion, and 10% ginger, the ash content was the highest. The increase in the ash content may be due to the high amount of red pepper and ginger because red pepper and ginger are a good source of minerals compared to garlic and red onion (Kefale et al., 2023), (Table 3). The values in this study were lower than the reported values for commercial red pepper paste (5.1%–21.1%) (Park et al., 2017). However, lower ash content (2.83%–3.1%) was also reported for red pepper paste that was prepared using a different milling technique (Bankole et al., 2013). With an increase in the mixing ratio of red pepper and garlic, the ash content of the product was increased (Figure 3). In contrast, increase in red onion and ginger significantly decreases the ash content of the paste, it may be due to the low ash content of red onion (Table 3).

**TABLE 3:** Physicochemical and Textural properties of red pepper, garlic, red onion, and ginger samples.

Parameters	Red pepper	Garlic	Red onion	Ginger
Color(a*)	32.51 ± 0.26^a^	5.94 ± 0.07^c^	6.58 ± 0.21^b^	3.07 ± 0.13
Ash content (%)	6.7 ± 0.28^a^	3.34 ± 0.05^d^	3.37 ± 0.019^b^	6.33 ± 0.25^c^
Fiber content (%)	38.77 ± 1.13^a^	3.03 ± 0.07^d^	3.87 ± 0.32^c^	6.62 ± 0.12^b^
Viscosity(cps)	45 ± 1.3^b^	27 ± 1.41^c^	42.5 ± 2.2^b^	311.5 ± 2.12^a^
Texture(hardness, g)	572.3 ± 6.49^ab^	860.033 ± 6.19 ^b^	675.83 ± 4.85^ab^	1516.66 ± 6.56^a^

*Note*: (Kefale et al., 2023). Values with the same letter in the same row are not significantly different at p<0.05. Color(a*)=redness value.

**TABLE 4:** Effect of red pepper, garlic, red onion, and ginger on color values of spicy red pepper paste (*Awaze*) and control sample (household made).

FM	Mixture component (%)				Color value				
	Red pepper (%)	Garlic (%)	Red onion (%)	Ginger (%)	Lightness (L*)	Redness (a*)	Yellowness (b*)	Chroma	Hue angle
1	60	20.22	12.01	7.77	13.8 ± 0.77^cd^	17.2 ± .49^bcde^	20.9 ± .85^abc^	27 ± .97^bc^	50.65 ± .33^a^
2	67.28	19.37	8.28	5.07	10.7 ± 0.49^cdef^	17.3 ± 1.06^bcde^	17.4 ± .92^bcdef^	24.5 ± 1.40^bcde^	45.14 ± .20^abcd^
3	70.13	10.00	9.87	10	13.5 ± 2.62^cd^	17.7 ± 1.06 ^bcde^	19.6 ± 1.70^bcd^	26.4 ± 1.97^bcd^	47.98 ± .73^ab^
4	68.84	13.99	12.16	5.01	18.5 ± 1.41^ab^	20.9 ± 1.34^ab^	22.4 ± 1.98^ab^	30.7 ± .53^ab^	47.01 ± 4.50^abc^
5	60	30.00	5.00	5	10.1 ± .92^def^	15.6 ± .28^cdef^	14.7 ± .92^cdef^	21.4 ± .83^cdef^	43.21 ± 1.29^abcde^
6	76.39	10.00	8.60	5.01	7.75 ± .92^fg^	14.7 ± 1.13^def^	11.6 ± 1.56^efg^	18.7 ± 1.85^def^	38.29 ± 1.50^de^
7	64.55	15.46	10.17	9.82	13.3 ± .64^cde^	17 ± .78^bcde^	18.3 ± 1.20 ^bcde^	24.9 ± 1.41^bcde^	47.06 ± .57^abc^
8	60	15.00	20.00	5	7.45 ± .21^fg^	14.2 ± .49^ef^	11.1 ± .21^efg^	18 ± .52^ef^	37.95 ± .50^de^
9	68.88	16.10	5.02	10	14.9 ± .28^bc^	17.2 ± .00^bcde^	20.1 ± .35^bcd^	26.4 ± .27^bcd^	49.36 ± .52^ab^
10	65.27	10.00	17.70	7.03	12.7 ± .57^cde^	18.3 ± .28^bcd^	18.3 ± .07^bcde^	25.9 ± .15^bcde^	44.85 ± .61^abcd^
11	74.39	13.33	5.00	7.28	19.9 ± 2.05^a^	23.4 ± 1.70^a^	28.1 ± 3.46^a^	36.5 ± 3.74^a^	50.05 ± 1.50^ab^
12	63.27	24.68	7.03	5.02	15 ± 1.27^bc^	19.3 ± 1.84^abc^	22.7 ± 4.88^ab^	29.8 ± 4.90^ab^	49.36 ± 3.43^ab^
13	60	10.13	19.87	10	8.95 ± .64^efg^	14 ± .28^ef^	14.1 ± 2.40^cdef^	19.9 ± 1.90^cdef^	44.98 ± 4.24^abcd^
14	64.15	20.85	5.00	10	8.05 ± .64^fg^	13.7 ± 1.13^ef^	12.6 ± 1.48^def^	18.6 ± 1.84^def^	42.44 ± 1.10^bcde^
15	60	14.81	15.20	9.99	5.4 ± .57^g^	11.6 ± 1.70^f^	9.7 ± 1.41^fg^	15.1 ± 2.2^fg^	40.03 ± .00^cde^
Control	HF	HF	HF	HF	5.3 ± .28^g^	5.85 ± .06^g^	4.15 ± .21^g^	7.17 ± .06^g^	35.35 ± 161^e^

Abbreviations: FM, formulations; HF, (household made formulation).L*, lightness, a*, redness, b*, yellowness.

*Note*: Data are means ± standard deviation of the replicates. Values with different lowercase letters in the same column are significantly different (LSD, *p* < .05).

**TABLE 5:** Effect of red pepper, garlic, red onion, and ginger on physicochemical property of formulated spicy red pepper paste (*Awaze*) and control sample (household made).

FM	Mixture component (%)				Physicochemical quality			
	Red pepper (%)	Garlic (%)	Red onion (%)	Ginger (%)	Ash (%)	Fiber (%)	Viscosity (cps)	RSA% at 20 μL
1	60	20.22	12.01	7.77	8.67 ± 0.22^de^	18.86 ± 0.52^h^	102 ± 1.4^cd^	54.13 ± 2.14^a^
2	67.28	19.37	8.28	5.07	6.89 ± 0.03^h^	24.22 ± 0.33^ef^	110 ± 2.83^bc^	42.44 ± 0.08^abcde^
3	70.13	10.00	9.87	10	6.87 ± 0.04^h^	14.60 ± 0.54^i^	121 ± 2.83^a^	48.48 ± 1.91^abcd^
4	68.84	13.99	12.16	5.01	8.25 ± 0.08^ef^	21.35 ± 0.21^g^	76.5 ± 2.12^ghi^	34.24 ± 1.07^e^
5	60	30.00	5.00	5	9.26 ± 0.06^bc^	18.68 ± 0.27^h^	111.5 ± 0.7^b^	48.49 ± 0.37^abcd^
6	76.39	10.00	8.60	5.01	8.80 ± 0.04^d^	29.06 ± 0.48^a^	89.5 ± 2.12^ef^	44.98 ± 0.8^abcde^
7	64.55	15.46	10.17	9.82	7.79 ± 0.03^g^	27.50 ± 0.01^abc^	125.5 ± 2.12^a^	48.13 ± 2.36^abcd^
8	60	15.00	20.00	5	7.90 ± 0.08^fg^	24.16 ± 0.46^ef^	93.5 ± 0.71^e^	51.10 ± 1.82^abc^
9	68.88	16.10	5.02	10	9.94 ± 0.06^a^	22.64 ± 0.47^fg^	91.5 ± 2.12^ef^	48.51 ± 0.96^abcd^
10	65.27	10.00	17.70	7.03	7.56 ± 0.07^g^	27.55 ± 0.61^abc^	104.5 ± 2.1^bc^	44.64 ± 1.18^abcde^
11	74.39	13.33	5.00	7.28	8.84 ± 0.02^cd^	27.90 ± 0.83^ab^	78.5 ± 2.12^i^	44.79 ± 1.61^abcde^
12	63.27	24.68	7.03	5.02	9.40 ± 0.03^b^	22.14 ± 0.23^g^	71 ± 1.41^gh^	40.54 ± 1.29^cde^
13	60	10.13	19.87	10	6.32 ± 0.05^i^	19.39 ± 0.10^h^	68.5 ± 0.71^hi^	33.56 ± 0.96^e^
14	64.15	20.85	5.00	10	7.86 ± 0.04^fg^	25.12 ± 0.28^de^	76.5 ± 2.12^ghi^	52.99 ± 2.68^ab^
15	60	14.81	15.20	9.99	7.81 ± 0.02^g^	26.58 ± 0.49^bcd^	95.5 ± 2.12^de^	39.14 ± 1.12^de^
Control	HF	HF	HF	HF	7.82 ± 0.308^g^	25.90 ± 0.14^cde^	84.5 ± 3.50^fg^	43.69 ± 1.44^abcde^

*Note*: Data are means ± standard deviation of the replicates. Values with different lowercase letters in the same column are significantly different (LSD, *p* < .05).

Abbreviations: FM, formulations; HF, (household‐made formulation); RSA, free radical scavenging activity.

**TABLE 6:** Correlation coefficients between ash content, Fe, Zn, K, Ca, and Mg contents of the formulations of *Awaze* paste.

	2	3	4	5	6
1. ash content	0.133	−0.052	0.105	−0.309	−0.132
2. Fe		0.66**	0.189	0.283	0.273
3. Zn			0.086	0.306	0.206
4. K				−0.447	0.843***
5. Ca					−0.310
6. Mg					

*Note*: *p < .05; **p < .01; ***p < .001.

**TABLE 7:** Effect of red pepper, garlic, red onion, and ginger on mineral contents (mg/100 g) of formulated spicy red pepper paste (*Awaze*) and control sample (household made).

FM	Mixture component (%)				Mineral content(mg/100 g)				
	Red pepper (%)	Garlic (%)	Red onion (%)	Ginger (%)	Fe	Zn	K	Ca	Mg
1	60	20.22	12.01	7.77	65.08 ± 0.14 ^d^	3.26 ± 0.32^g^	2291.3 ± 1.28^h^	45.67 ± 0.8^e^	174.85 ± 0.63^g^
2	67.28	19.37	8.28	5.07	47.62 ± 0.59 ^h^	12.25 ± 0.12^b^	2367.17 ± 1.16^f^	63.7 ± 0.53^f^	169.55 ± 0.49^c^
3	70.13	10.00	9.87	10	61.43 ± 051 ^f^	11.42 ± 0.08^c^	2496.22 ± 0.27^e^	49.32 ± 0.33^b^	194.62 ± 0.59^f^
4	68.84	13.99	12.16	5.01	49.22 ± 011 ^h^	4.52 ± 0.03^f^	2191.39 ± 0.04^k^	40.41 ± 0.24^i^	157.08 ± 0.078^j^
5	60	30.00	5.00	5	35.59 ± 012^k^	1.86 ± 0.09^jk^	2270.82 ± 0.01^i^	42.59 ± 0.2^k^	143.37 ± 0.76^hi^
6	76.39	10.00	8.60	5.01	108.82 ± 0.82^a^	26.93 ± 0.16^a^	2314.15 ± 0.31^g^	59.24 ± 0.11^e^	174.79 ± 0.04^d^
7	64.55	15.46	10.17	9.82	62.82 ± 0.02^ef^	8.57 ± 0.27^d^	2533.27 ± 0.06^d^	72.27 ± 0.09^c^	192.14 ± 0.32^b^
8	60	15.00	20.00	5	73.83 ± 0.01^b^	3.11 ± 0.08^gh^	2556.17 ± 0.14^c^	43.61 ± 0.41^e^	176.01 ± 0.06^h^
9	68.88	16.10	5.02	10	63.36 ± 0.64^e^	5.6 ± 0.12^e^	2142.92 ± 0.10^L^	28.09 ± 0.02^g^	164.2 ± 0.05^n^
10	65.27	10.00	17.70	7.03	45.37 ± 0.74^i^	1.72 ± 0.09^k^	1991.77 ± 0.2^m^	42.2 ± 0.14^c^	132.66 ± 0.55^i^
11	74.39	13.33	5.00	7.28	59.61 ± 0.07^g^	3.59 ± 0.12^g^	2580.9 ± 0.07^b^	35.6 ± 0.05^h^	161.29 ± 0.22^l^
12	63.27	24.68	7.03	5.02	71.91 ± 0.08^c^	2.5 ± 0.01^hi^	2783.32 ± 0.01^a^	58.07 ± 0.01^d^	186.81 ± 0.20^d^
13	60	10.13	19.87	10	72.34 ± 0.11^bc^	3.58 ± 0.05^g^	2313.4 ± 0.21^g^	54.28 ± 0.1^a^	198.68 ± 0.23^e^
14	64.15	20.85	5.00	10	36.54 ± 0.47^k^	3.51 ± 0.06^g^	2190.82 ± 0.06^k^	38.49 ± 0.43^c^	192.12 ± 0.72^k^
15	60	14.81	15.20	9.99	41.51 ± 0.29 ^j^	2.43 ± 0.08^ij^	2256.32 ± 0.10^j^	32.12 ± 0.05^j^	148.13 ± 0.57^m^
Control	HF	HF	HF	HF	58.68 ± 0.48^g^	4.63 ± 0.13^f^	913.34 ± 0.19^n^	92.27 ± 013^m^	73.48 ± 0.55^a^

*Note*: Data are means ± standard deviation of the replicates. Values with different lowercase letters in the same column are significantly different (LSD, *p* < .05).

Abbreviations: FM, formulations; HF, (household made formulation).

**TABLE 8:** Effect of red pepper, garlic, red onion, and ginger on texture profile of formulated spicy red pepper paste (*Awaze*) and control sample (household made).

FM	Mixture component (%)				Texture profile					
	Red pepper (%)	Garlic (%)	Red onion (%)	Ginger (%)	Hardness (g)	Adhesiveness (g.s)	Cohesiveness	Springiness	Gumminess (g)	Chewiness(g)
1	60	20.22	12.01	7.77	8.88 ± .19^fg^	−6.06 ± .36^a^	.68 ± .06^ab^	0.95±. 007^b^	6.63 ± .16^b^	6.30 ± .10 ^bc^
2	67.28	19.37	8.28	5.07	8.48 ± .37^g^	−6.56 ± .08^abc^	.78 ± .02^a^	0.88 ± .03^bc^	7.39 ± .30^b^	6.54 ± .52^c^
3	70.13	10.00	9.87	10	13.36 ± .77^def^	−7.49 ± .35^abcd^	.57 ± .01^bcd^	0.5 ± .028^def^	5.23 ± .28^b^	2.63 ± .30^c^
4	68.84	13.99	12.16	5.01	11.35 ± .64^efg^	−6.29 ± .73^ab^	.56 ± .04 ^bcd^	0.7 ± .1^bcde^	6.50 ± 1.54^b^	4.70 ± 2.02^c^
5	60	30.00	5.00	5	13.62 ± 1.22^de^	−8.55 ± .47^bcd^	.63 ± .02 ^abc^	0.7 ± 0.007^bcde^	7.90 ± .25^b^	5.54 ± .69^c^
6	76.39	10.00	8.60	5.01	14.43 ± .69^de^	−9.07 ± .47^d^	.63 ± .06^abc^	0.7 ± 0.007^bcde^	8.43 ± 1.41^ab^	6.03 ± 1.65^c^
7	64.55	15.46	10.17	9.82	10.11 ± .55^efg^	−6.31 ± .21^ab^	.63 ± .05^abcd^	0.83 ± .007^bcd^	6.95 ± .53^b^	5.82 ± .52^c^
8	60	15.00	20.00	5	16.33 ± .04^d^	−7.78 ± 1.21^abcd^	.48 ± .08^de^	1.5 ± .0.03^a^	11.69 ± .72^ab^	17.62 ± 1.44^a^
9	68.88	16.10	5.02	10	12.57 ± .13^defg^	−7.37 ± .27^abcd^	.59 ± .03^bcd^	0.53 ± .08^cdef^	5.23 ± 1.17^b^	2.84 ± 1.08^c^
10	65.27	10.00	17.70	7.03	23.59 ± 1.29^c^	−13.15 ± .30^ef^	.56 ± .04^bcd^	0.54 ± 0.10^cdef^	11.89 ± 1.93^ab^	6.57 ± 2.25^bc^
11	74.39	13.33	5.00	7.28	28.68 ± 2.08^b^	−16.34 ± .73^g^	.57 ± .01^bcd^	0.41 ± 0.12^ef^	10.20 ± 2.63 ^ab^	4.34 ± 2.37^c^
12	63.27	24.68	7.03	5.02	25.41 ± 1.73^bc^	−14.01 ± 1.28^f^	.55 ± .01^bcd^	0.76 ± 0.07^bcd^	17.34 ± 2.69^a^	13.41 ± 3.45^ab^
13	60	10.13	19.87	10	21.71 ± .24^c^	−11.48 ± .30^e^	.53 ± .02^cde^	0.64 ± .02^bcde^	11.01 ± .39^ab^	7.05 ± .56^bc^
14	64.15	20.85	5.00	10	15.96 ± .90^d^	−8.66 ± .07^cd^	.55 ± .02^bcde^	0.62 ± .17^bcde^	7.58 ± 2.83^b^	4.96 ± 3.10^c^
15	60	14.81	15.20	9.99	55.09 ± 2.78^a^	−21.77 ± .22^h^	.40 ± .02^e^	0.22 ± 0.14^f^	10.79 ± 6.83^ab^	2.87 ± 3.04^c^
Control	HF	HF	HF	HF	14.12 ± .06^de^	−7.34 ± .30^abcd^	.52 ± .03^cde^	0.63 ± .0.02^bcde^	5.34 ± .05^b^	3.41 ± .15^c^

Abbreviations: FM, formulations; HF, (household made formulation).

*Note*: Data are means ± standard deviation of the replicates. Values with different lowercase letters in the same column are significantly different (LSD, *p* < .05).

**TABLE 9:** The predicted models to experimental data in D‐optimal mixtures design for the dependent variables of spicy red pepper paste (*Awaze*).

Response	Model	*F*‐value	Lack of fit (*p* > .05)	*R* ^2^
Ash content	linear	2.73	.0944	.42
Chewiness	linear	4.23	.0323	.535
Color (a*)	quadratic	2.88	.1571	.83
Percentage of free radical scavenging activity	quadratic	2.66	.1118	.82
Fiber content	Special cubic	6.44	.2366	.98

*Note*: a*, redness value.

**TABLE 10:** Predicted and verified values of spicy red pepper paste (*Awaze*) optimized formula.

Response	Optimum formula						Desirability
	65.66% red pepper +10% garlic +19.086% red onion+5.254% ginger						
	Predicted value	Verified value	Low 95% CI	High 95% CI	Low 95% PI	High 95% PI	
Color (a*)	21.57	18.30	17.96	25.17	15.23	27.91	0.561
Antioxidant activity (%)	44.31	44.64	36.64	51.99	30.83	57.80	
Viscosity	94.37	104.50	84.42	104.31	54.58	134.15	
Ash content (%)	8.30	7.56	7.77	8.83	6.32	10.28	
Fiber content (%)	24.03	27.55	2.34	50.40	10.02	58.08	
Chewiness (g)	6.88	6.57	4.99	8.77	0.16	13.93	

*Note*: a*, redness value.

### 3.3 Effect of red pepper, garlic, red onion, and ginger on crude fiber content of *awaze* paste

**FIGURE 4:**
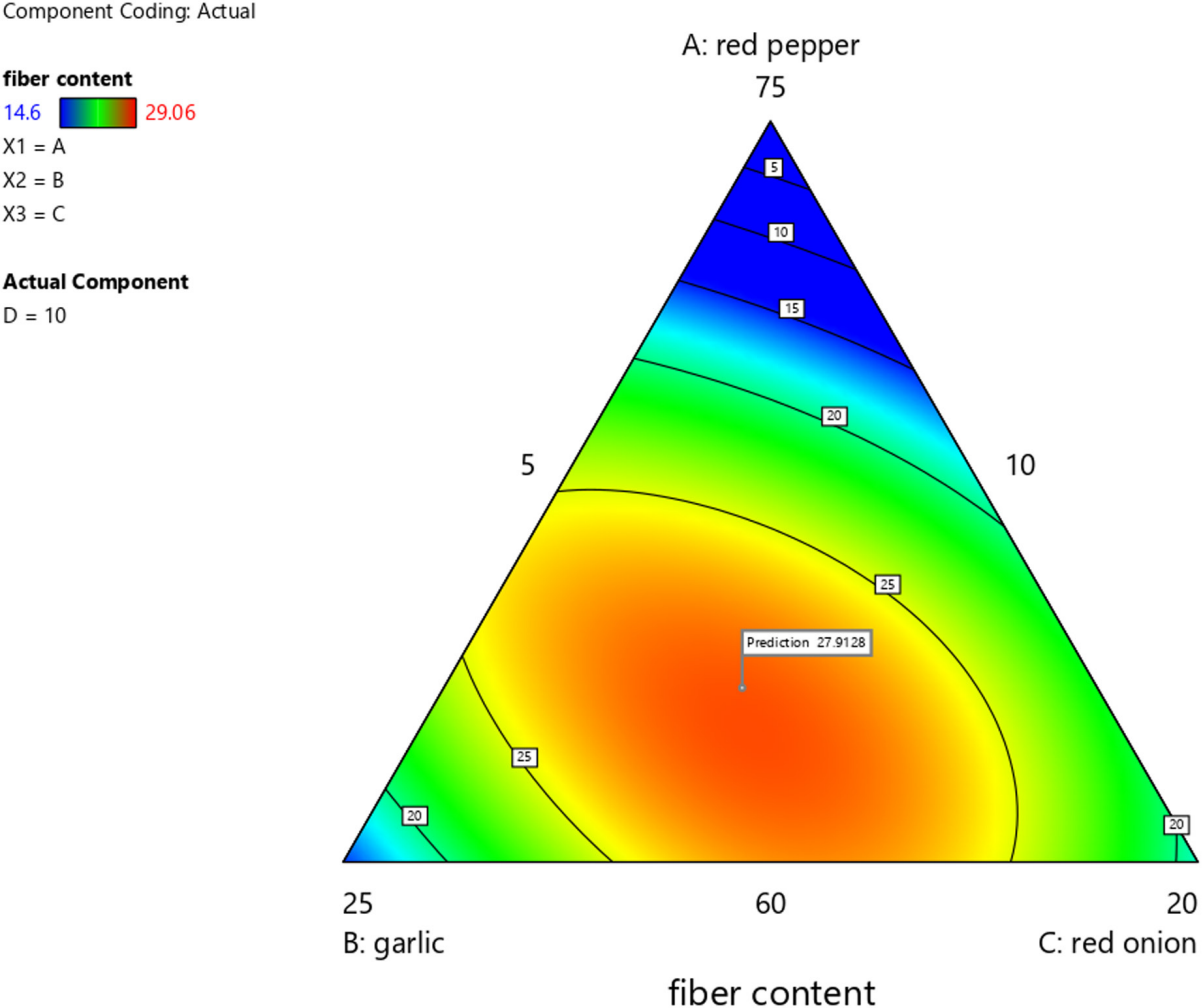
The effect of blending ratio on fiber content of spicy red pepper paste (*Awaze)* obtained using actual components as shown by contour plot.

The blending ratios resulted in a significant change (*p* < .05) in fiber content. The crude fibers of the formulated *Awaze* paste varied from 16.86 to 29.06% (Table 5). The crude fiber content of red pepper, garlic, red onion, and ginger (individual samples) was 38.77%, 3.03%, 3.87%, 6.62%, respectively, as shown in Table 3. The maximal values for fiber content (29.06%) were found for formulation with 76.39% red pepper, 10% garlic, 8.6% red onion, and 5.01% for ginger, whereas the lowest fiber content (14.6%) is identified in *Awaze* paste from 70.13% red pepper, 10% garlic 9.87% red onion, and 10% ginger. The variation in the fiber content of the formulations is due to the difference in the blending ratio of the ingredients (spices).The crude fiber content found in this study was higher compared to the reported values of sweet red pepper paste(3.61%) (Dimassi et al., 2019). The crude fiber content of formulated *Awaze* paste was higher (16.86%–29.06%) than study report on cheese supplemented sweet red pepper paste (Atwaa et al., 2020). Based on the contour plot, the fiber content of *Awaze* paste was increased with an increase in mixing ratio of red pepper, red onion, and ginger as shown in Figure 4.Therefore, higher dietary fiber content is similarly expected with an increasing ratio of red pepper, ginger, and red onion used in this study, which have higher fiber content and contributed for higher fiber as their proportion increased in the formulation.

### 3.4 Effect of red pepper, garlic, red onion, and ginger proportion on viscosity *of Awaze* paste

The result indicated that the viscosity of the formulated *Awaze* paste was significantly different at *p* < .05 (Table 5).Viscosity is an important parameter for texture of food products including food and condiment pastes. The viscosity of the formulations and control sample was in the range of 71–125.5 cps. The maximal viscosity was found to be at a mixing ratio of 64.55% red pepper, 15.46% garlic, 10.17% red onion, and 9.82% for ginger. Besides, the minimal values were found to be 63.27%, 24.68%, 7.03%, and 5.02% in the corresponding order. This could be due to the relatively higher ratio of ginger and red onion because ginger and red onion had higher viscosity compared to red pepper and garlic (Kefale et al., 2023) (Table 3). The viscosity values found in this study are lower compared to the reported values of onion paste (396–398 cps) (Naqash et al., 2021). Also, the lower viscosity (13.24–85.84 cps) was reported for hot pepper‐soya bean paste at different solid contents and temperatures (Yo, 2001). A previous study reported the viscosity of salad dressing in the range of 14.3–18.1 cps (Koc, 2020) which is lower compared to the current result. It may be the difference in ingredient types used.

### 3.5 Effect of red pepper, garlic, red onion, and ginger on antioxidant activity of *Awaze* paste

The blending ratio resulted in a significant change at *p* < .05 in the antioxidant activity of the formulated *Awaze* paste. Total antioxidant activity is an important parameter in establishing the health functionality of spices and vegetable product. The antioxidant activity of the formulated product (free radical scavenging activity towards DPPH) was observed at 20 μL concentration (Table 5). The antioxidant activity (free radical scavenging activity towards DPPH) was in the range of 34.24%–54.13% (% inhibition) for all formulations at a concentration of 20 μL. The highest antioxidant activity of 54.13% was found for formulations that contained 60% red pepper, 20.2% garlic, 12.01% red onion, and 7.77% ginger. The lowest antioxidant activity of 34.24% was found for formulations that contained 68.84% red pepper, 13.99% garlic, 12.12% red onion, and 5.01% ginger, it may be due to the relatively low proportion of garlic in the formulation. This report is in agreement with the study that reported an antioxidant activity in the range of 30%–90% for different spices at different concentrations (Sasikumar et al., 2020). A previous study obtained an antioxidant activity of hot and sweet red pepper paste in the range 40%–90% (Sayin & Arslan, 2015), which is higher than the result of the current study. A previous study reported on DPPH activity of soya bean‐based red pepper paste in the range 31.88%–43.19% on different fermentation times (Yang et al., 2018), which is lower antioxidant activity content compared to the current result.

**FIGURE 5:**
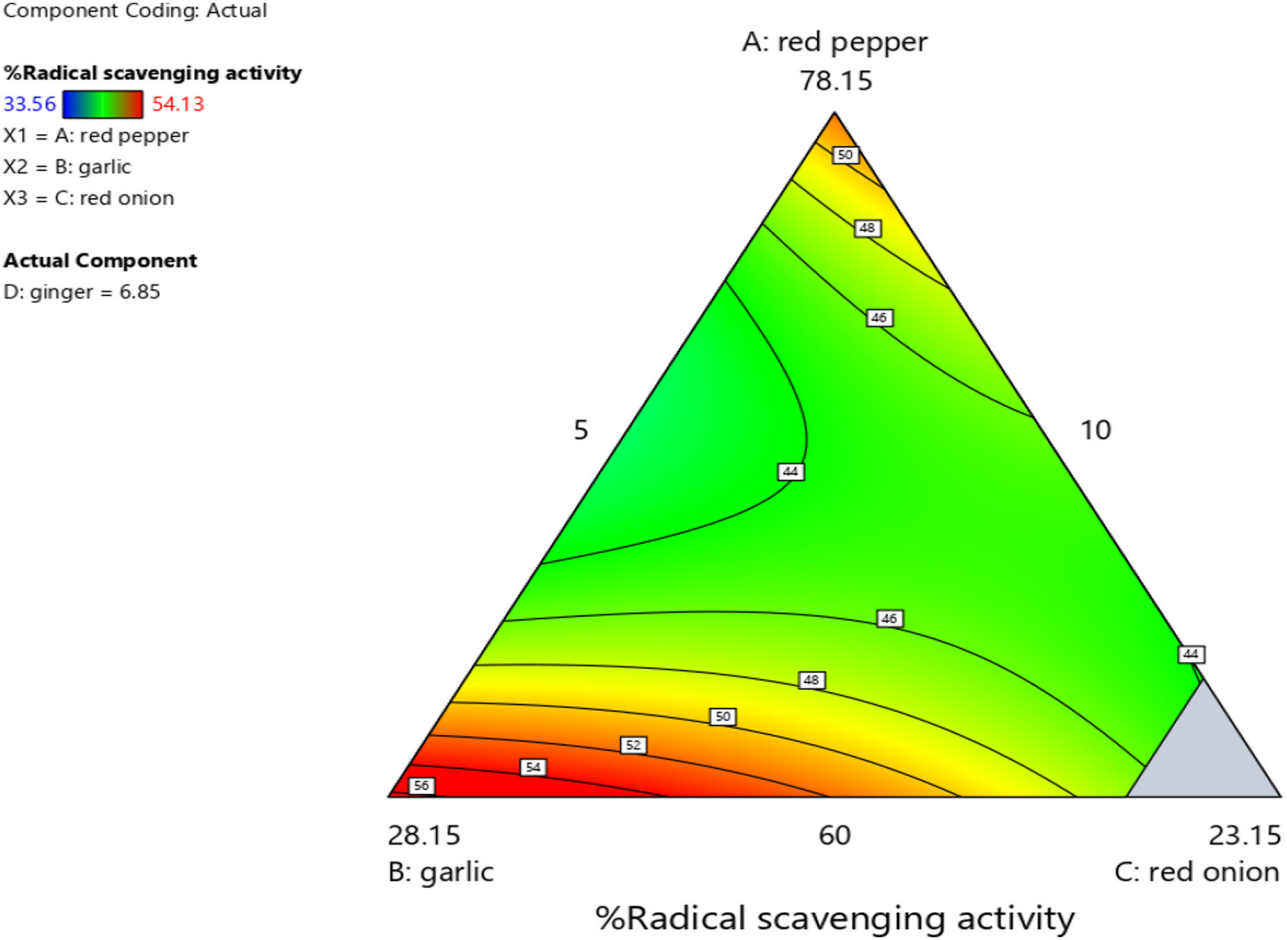
The effect of blending ratio on % free radical scavenging activity of spicy red pepper paste (*Awaze)* obtained using actual components as shown by contour plot.

As shown in Figure 5, as the mixing ratio of red pepper and ginger increases, the percentage of free radical scavenging was significantly increased. As the mixing ratio of red onion was increased, the percentage of free radical scavenging was decreased. The percentage of free radical was increased with an increase in the ratio between garlic and red pepper. Therefore, we suggest that a significant difference of antioxidant activity *Awaze* pastes is due to the difference in mixing ratio of ingredients.

### 3.6 Effect of red pepper, garlic, red onion, and ginger proportion on mineral contents of *awaze* paste

The result indicated a significant change in mineral contents (Fe, Zn, K, Ca, and Mg) for the formulations and control sample (*p* < .05) (Table 7).The formulated spicy red pepper paste (*Awaze*) had mean values of Fe, Zn, K, Ca, and Mg in the range of 36.54–108.82, 1.72–26.93, 913.34–2783.32,28.09–92.27, and 73.48–198.6 mg/100 g, respectively. Formulation of 76.39% red pepper, 10% garlic, 8.6% red onion, and 5.01% ginger had highest Fe and Zn content of 108.82 and 26.93 mg/100 g, respectively, this may be due to the high proportion of red pepper and red onion. Formulation of 63.27% red pepper, 24.68% garlic, 7.03% red onion, and 5.02% ginger had highest K content with the value 2783.32 mg/100 g that is due to the high proportion of red onion. The control sample had higher Ca content 92.27 mg/100 g and lower K content (913.4 mg/100 g) and Mg content (73.5 mg/100 g) compared to the formulations of this study.

At a mixing ratio of 60% red pepper, 10.13% garlic, 19.87% red onion, and 10% ginger, the concentration of Mg had the highest value (198.68 mg/100 g). A previous study on red paper paste reported Fe content in the range of 13–35 mg/100 g and zinc content in the range of 3.3–10 mg/100 g (Park et al., 2017) which is lower than the results of the current study. These findings imply Fe and Zn content in the formulated paste is a good input for the food industry that produces condiment‐based paste products.

#### 3.6.1 Correlation between ash content, Fe, Zn, K, Ca, and Mg contents of the formulations of *Awaze* paste

According to Pearson correlation test, Table 6 demonstrates the correlation coefficients between ash content and minerals (Fe, Zn, K, Ca, and Mg). Highly significant correlation coefficients were found between K and Mg (*r* = .843), Fe and Zn (*r* = .66) (*p* < .05). No statically significant positive correlation coefficient was found between ash content and Fe (*r* = .133), ash content and K (*r* = .105), Fe and K (*r* = .189), Zn and K (*r* = .086), Fe and Ca (*r* = .28), Zn and Ca (*r* = .306), Fe and Mg (*r* = .27), Zn and Mg (0.206) (*p* > .05). No statically significant negative correlation coefficients were found between K and Ca, Ca and Mg, ash content and Zn, Ca, Mg content of the formulated *Awaze* paste (*p* > 0.05). Depending on several variables, such as raw materials, processing condition, and formulation difference which interact with each other, because of all these interaction factors affecting the correlation of ash content and mineral content of the formulated *Awaze* paste.

### 3.7 Effect of red pepper, garlic, red onion, and ginger proportion on texture profile of *Awaze* paste

The textural property of *Awaze* paste is shown in Table 8. The result showed that the blending ratio of the four ingredient powders (red pepper, garlic, red onion, and ginger) had a significant (*p* < .05) effect on the textural property of *Awaze* paste. Hardness value of the formulation with 60% red pepper, 14.81% garlic, 15.2% red onion, and 9.9% ginger had the highest value (55.09 g) that was due to the highest proportion of garlic and ginger. Cohesiveness indicates the strength of the internal bonds in making of *Awaze* paste. As seen from Table 6, cohesiveness of the formulation was in the range of 0.40–0.78. The highest cohesiveness score was obtained for formulation with 67.28% red pepper, 19.37% garlic, 8.28% red onion, 5.05% ginger which contained relatively high proportion of red pepper and garlic.

The range of springiness score of the formulations was 0.22–1.5. Formulation with 60% red pepper, 15% garlic, 20% red onion, and 5.0% ginger had the highest springiness score (1.5) that corresponded to high proportions of red pepper and ginger during preparation of *Awaze* paste. Gumminess score of the formulation were in the range of 5.23–17.32 g, whereas the highest gumminess value was obtained for formulation with 63.27% red pepper, 24.68% garlic, 7.03% red onion, and 5.02% ginger which was prepared from high proportion of red onion and ginger. Chewiness is a function of hardness. As seen from Table 6, chewiness score of formulation with 60% red pepper, 15% garlic, 20% red onion, and 5% ginger was the highest (17.62 g).

The current result of hardness, cohesiveness, gumminess, springiness, and chewiness had lower value compared to the reported value on freeze dried fermented chili paste (Man et al., 2022). The freeze drying process influences the texture profile of the product. It is because the lyophilization of fermented chili powder was extremely dry, whereas fermented chili paste has a humidity of approximately 90%. A previous study report on the texture profile for miser paste observed higher values (Ergönül, 2013), compared to the current result. Generally, interaction between red pepper, garlic, red onion, and ginger had an effect on the texture profile of *Awaze* paste.

### 3.8 Model development

Independent and dependent variables were fitted to linear, quadratic, and special cubic models (Table 9). To check goodness of model fit, residual plots were created. The best model has low standard deviation, high predicted R‐squared (R^2^), low predicted sum of square, and lack of fit (*p* > .05) (Nikzade et al., 2012). Linear model was found to be the best fit for ash content and chewiness. Quadratic model was found to be the best fit for color (a*) and free radical scavenging activity. Special cubic model was found to be the best fit for fiber content (*R* ^2^ = .98) (Table 9).

### 3.9 Optimization

An optimization of *Awaze* paste formulation was done using the method by Derringer & Suich, 1980. The optimization was done to develop a high‐quality *Awaze* paste with the selected important parameters, including color (a* value), antioxidant activity, viscosity, ash content, fiber content, and chewiness (Table 10). During numerical optimization an attempt was made to maximize all the response. The ingredient red pepper, garlic and ginger was set to the range while red onion was set to the maximum. High relative importance of “5” was assigned to antioxidant activity and color (a*). High relative importance of “3” was assigned to ash content. High relative importance of “2” was assigned to viscosity, fiber content, and chewiness. The numerical optimization showed that *Awaze* paste made from 65.66% red pepper, 10% garlic, 19.086% red onion, and 5.254% ginger achieved the best desirability (0.561).The predicted response values for color(a* value), viscosity, ash, fiber, chewiness, and antioxidant activity for this optimal formulation were 21.56, 94.36 cps, 8.3%, 24.03%, 6.88 g, and 44.3%, respectively (Table 10).

### 3.10 Verification of the model

**FIGURE 6:**
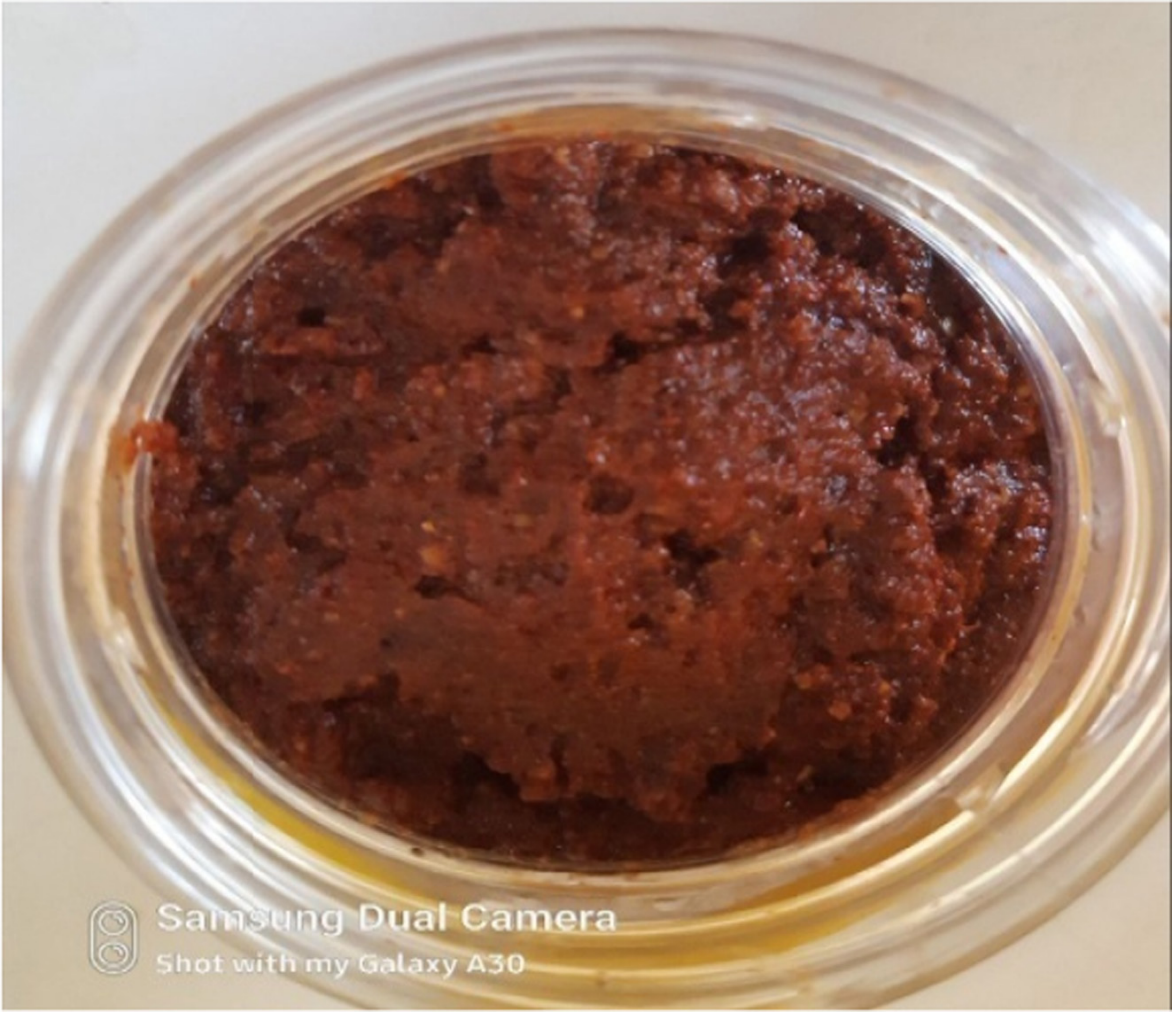
*Awaze* paste prepared from red pepper, garlic, red onion, and ginger by using the optimum formula.

The predicted optimum formulation was verified using an independent real formulation study. The comparison between the predicted (expected) and real values of *Awaze* paste for the selected parameters are shown in Table 10. The real product is shown in Figure 6. The predicted and verified values of the analyzed responses did not differ statistically as the verified values were included in 95% confidence interval and prediction interval range. Simultaneous optimization of *Awaze* paste was done using numerical and graphical optimization, where desirability values can range between 0 and 1. Zero indicates no desirability and 1 indicates higher possible desirability with respect to the constraints imposed. In this study, 6 dependent parameters, which are very important quality indicators, were selected for optimization study and the constrains are listed subsequently. The results were shown in Table 10, from this result it can be clearly predicted that *Awaze* paste can be prepared with optimum quality attributes using 65.66% red pepper, 10% garlic, 19.086% red onion, and 5.254% ginger with desirability of 0.56.

## 4 CONCLUSION

D‐optimal mixture design was used to optimize the formulation of functional *Awaze* by red pepper, garlic, red onion, and ginger. Formulation of *Awaze* with red pepper, garlic, red onion, and ginger resulted in a product with higher antioxidant content and better nutritional characteristics compared to the control sample. In the optimal sample, the redness (a*) value increased by more than 3.12 times and the viscosity value also by more than by 20 compared to the control. The formulated *Awaze* paste had higher antioxidant activity, ash content, fiber content, color value (a*), viscosity, and chewiness compared to the control (household‐made *Awaze* paste) sample. Based on our results, red pepper, garlic, red onion, and ginger have the potential to be used as an added valuable supplement in *Awaze* production to improve the nutritional properties.

## AUTHOR CONTRIBUTIONS

**Biadge Kefale:** Conceptualization (equal); data curation (equal); formal analysis (equal); funding acquisition (equal); investigation (equal); methodology (equal); project administration (equal); resources (equal); software (equal); supervision (equal); validation (equal); visualization (equal); writing – original draft (equal); writing – review and editing (equal). **Mulugeta Admasu Delele:** Conceptualization (equal); data curation (equal); formal analysis (equal); funding acquisition (equal); investigation (equal); methodology (equal); project administration (equal); resources (equal); software (equal); supervision (equal); validation (equal); visualization (equal); writing – original draft (equal); writing – review and editing (equal). **Solomon Workneh Fanta:** Conceptualization (equal); data curation (equal); formal analysis (equal); funding acquisition (equal); investigation (equal); methodology (equal); project administration (equal); resources (equal); software (equal); supervision (equal); validation (equal); visualization (equal); writing – original draft (equal); writing – review and editing (equal). **Solomon Abate Mekonnen:** Conceptualization (equal); data curation (equal); formal analysis (equal); funding acquisition (equal); investigation (equal); methodology (equal); project administration (equal); resources (equal); software (equal); supervision (equal); validation (equal); visualization (equal); writing – original draft (equal); writing – review and editing (equal).

## CONFLICT OF INTEREST STATEMENT

There is no conflict of interest.

## CONSENT FOR PUBLICATION

All author consent for publication.

## Data Availability

Data will be available upon request.

## References

[fsn33874-bib-0001] Aggarwal, A. , Rehm, C. D. , Monsivais, P. , & Drewnowski, A. (2016). Importance of taste, nutrition, cost and convenience in relation to diet quality: Evidence of nutrition resilience among US adults using National Health and nutrition examination survey (NHANES) 2007–2010. Preventive Medicine, 90, 184–192. 10.1016/j.ypmed.2016.06.030 27374943 PMC5303533

[fsn33874-bib-0002] Al Hagbani, T. , & Nazzal, S. (2018). Medicated chewing gums (MCGs): Composition, production, and mechanical testing. AAPS PharmSciTech, 19(7), 2908–2920. 10.1208/s12249-018-1123-z 30088152

[fsn33874-bib-0003] AOAC . (1990). Official methods of analysis of the association of offical analytical chemists (15th ed.). (Volume 1), 73–80. (2003). Association of Official Analytical Chemist. 10.7312/seir17116-004

[fsn33874-bib-0004] Atwaa, E. , Ramadan, M. , & Abd El‐Sattar, E. (2020). Production of functional spreadable processed cheese supplemented with sweet red pepper paste. Journal of Food and Dairy Sciences, 11(5), 127–132. 10.21608/jfds.2020.102741

[fsn33874-bib-0005] Bankole, Y. O. , Taninola, O. A. , Adesina, B. S. , & Samuel, D. O. (2013). Quality Assessment of pepper paste using different milling methods. Academic Journal of Interdisciplinary Studies, 2(10), 187–192. 10.5901/ajis.2013.v2n10p187

[fsn33874-bib-0006] Cardoso, C. M. L. , Mendes, R. , & Nunes, M. L. (2009). Instrumental texture and sensory characteristics of cod frankfurter sausages. International Journal of Food Properties, 12(3), 625–643. 10.1080/10942910801992959

[fsn33874-bib-0007] Chen, J. , & Rosenthal, A. (2015). Food texture and structure. In Modifying food texture: Novel ingredients and processing techniques (Vol. 1). Elsevier Ltd. 10.1016/B978-1-78242-333-1.00001-2

[fsn33874-bib-0008] Demeke, D. , Getahun, B. , & Mulualem Atinafu, D. (2020). Assessing Ethnophysiological use of spices and condiments in Bahir Dar City market, Bahir Dar, Ethiopia: Ethnophysiological qualitative study. Biomedical Science, 6(2), 25. 10.11648/j.bs.20200602.12

[fsn33874-bib-0009] Derringer, G. , & Suich, R. (1980). Simultaneous optimization of several response variables. Journal of Quality Technology, 12(4), 214–219. 10.1080/00224065.1980.11980968

[fsn33874-bib-0010] Dessalegn, A. , & Ashenafi, M. (2010). Internet journal of food safety evaluation of the probiotic properties and antibiotic resistance of lactic acid bacteria isolated from awaze. Qotchqotcha and Tef Dough , Traditional Ethiopian Fermented Foods, 12(January 2010), 187–191.

[fsn33874-bib-0011] Dessie, A. B. , Koye, T. D. , Koye, A. D. , & Abitew, A. A. (2019). Analysis of red pepper marketing: Evidence from Northwest Ethiopia. Journal of Economic Structures, 8(1), 1–14. 10.1186/s40008-019-0156-0

[fsn33874-bib-0012] Dimassi, O. , Fakih, Y. , Sharif‐Askari, E. , Rached, M. , & Akiki, R. (2019). Drying dynamics, physicochemical properties and sensory analysis of sweet red pepper paste. International Journal of Horticulture, Agriculture and Food Science, 3(4), 216–223. 10.22161/ijhaf.3.4.10

[fsn33874-bib-0013] Dini, I. , Tenore, G. C. , & Dini, A. (2008). Chemical composition, nutritional value and antioxidant properties of allium caepa L. Var. tropeana (red onion) seeds. Food Chemistry, 107(2), 613–621. 10.1016/j.foodchem.2007.08.053

[fsn33874-bib-0014] Ergönül, B. (2013). Instrumental textural properties of mesir paste kept at different temperatures. International Journal of Food Properties, 16(7), 1530–1533. 10.1080/10942912.2011.599092

[fsn33874-bib-0015] Haciseferoǧullari, H. , Özcan, M. , Demir, F. , & Çalişir, S. (2005). Some nutritional and technological properties of garlic (*Allium sativum* L.). Journal of Food Engineering, 68(4), 463–469. 10.1016/j.jfoodeng.2004.06.024

[fsn33874-bib-0016] Hewlings, S. J. , & Kalman, D. S. (2017). Curcumin: A review of its effects on human health. Food, 6(10), 1–11. 10.3390/foods6100092 PMC5664031 29065496

[fsn33874-bib-0017] Idris, A. , Mehari, T. , & Ashenafi, M. (2001). Some microbiological and biochemical studies on the fermentation of “awaze” and “datta”, traditional Ethiopian condiments. International Journal of Food Sciences and Nutrition, 52(1), 5–14. 10.1080/09637480020027174 11225177

[fsn33874-bib-0018] Jang, M. S. , Park, J. E. , & Park, H. Y. (2011). Formulation optimization of salad dressing added with Chinese quince (Chaenomelis sinensis) juice by mixture design. Food Science and Biotechnology, 20(2), 409–417. 10.1007/s10068-011-0058-x

[fsn33874-bib-0019] Kefale, B. , Delele, M. A. , Fanta, S. W. , & Abate, S. M. (2023). *Properties of Red Pepper (Capsicum annuum L.), Red Onion (Allium cepa), Ginger (Zingiber officinale), and Garlic (Allium sativum): Main Ingredients for the Preparation of Spicy Foods in Ethiopia*. *2023* .

[fsn33874-bib-0020] Koc, G. C. (2020). Physicochemical, reconstitution, and morphological properties of red pepper juice (*Capsicum annuum* L.) powder. Journal of Food Science and Technology, 58, 4011–4023. 10.1007/s13197-020-04864-x 34471325 PMC8357926

[fsn33874-bib-0021] Lanzotti, V. (2006). The analysis of onion and garlic. Journal of Chromatography A, 1112(1–2), 3–22. 10.1016/j.chroma.2005.12.016 16388813

[fsn33874-bib-0022] Li, J. , Zhao, F. , Liu, H. , Li, R. , Wang, Y. , & Liao, X. (2016). Fermented minced pepper by high pressure processing, high pressure processing with mild temperature and thermal pasteurization. Innovative Food Science and Emerging Technologies, 36, 34–41. 10.1016/j.ifset.2016.05.012

[fsn33874-bib-0023] Luthria, D. L. , Mukhopadhyay, S. , & Krizek, D. T. (2006). Content of total phenolics and phenolic acids in tomato (Lycopersicon esculentum mill.) fruits as influenced by cultivar and solar UV radiation. Journal of Food Composition and Analysis, 19(8), 771–777. 10.1016/j.jfca.2006.04.005

[fsn33874-bib-0024] Man, S. , Chis, M. S. , Rusu, I. E. , & Vis, V. (2022). Freeze‐Dried Powder of Fermented Chili Paste—New Approach. 10.3390/foods11223716 PMC9689597 36429308

[fsn33874-bib-0025] Mao, Q. Q. , Xu, X. Y. , Cao, S. Y. , Gan, R. Y. , Corke, H. , Beta, T. , & Li, H. B. (2019). Bioactive compounds and bioactivities of ginger (zingiber officinale roscoe). Food, 8(6), 1–21. 10.3390/foods8060185 PMC6616534 31151279

[fsn33874-bib-0026] Mo, H. , Sung, J. , Su, K. , Hwang, J. E. , Jeon, S. G. , & Kim, B. S. (2015). Horticultural and chemical quality characterization of accessions selected from four species of capsicum. Horticulture Environment and Biotechnology, 56(1), 54–66. 10.1007/s13580-015-0078-0

[fsn33874-bib-0027] Naqash, S. , Naik, H. R. , Hussain, S. Z. , Makroo, H. A. , & Dar, B. N. (2021). Effect of thermal treatment on physicochemical, phytochemical, and microbiological characteristics of brown Spanish onion paste. Quality Assurance and Safety of Crops & Foods, 13(4), 1–12. 10.15586/qas.v13i4.959

[fsn33874-bib-0028] Nikzade, V. , Tehrani, M. M. , & Saadatmand‐Tarzjan, M. (2012). Optimization of low‐cholesterol‐low‐fat mayonnaise formulation: Effect of using soy milk and some stabilizer by a mixture design approach. Food Hydrocolloids, 28(2), 344–352. 10.1016/j.foodhyd.2011.12.023

[fsn33874-bib-0029] Oh, S. H. , Hwang, I. G. , Kim, H. Y. , Hwang, C. R. , Park, S. M. , & Hwang, Y. (2011). Quality characteristics by particle size of red pepper powders for pepper paste and kimchi. Journal of the Korean Society of Food Science and Nutrition, 40(5), 725–730. 10.3746/jkfn.2011.40.5.725

[fsn33874-bib-0030] Park, J. N. , Park, J. G. , Han, I. J. , Song, B. S. , Choi, J. I. , Kim, J. H. , Sohn, H. S. , & Lee, J. W. (2010). Combined effects of heating and γ‐irradiation on the microbiological and sensory characteristics of gochujang (Korean fermented red pepper paste) sauce during storage. Food Science and Biotechnology, 19(5), 1219–1225. 10.1007/s10068-010-0174-z

[fsn33874-bib-0031] Park, S.‐Y. , Kim, S. , Hong, S. , & Lim, S.‐D. (2017). Analysis of quality characteristics of traditional and commercial red pepper pastes (gochujang). Korean Journal of Food & Cookery Science, 33(2), 137–147. 10.9724/kfcs.2017.33.2.137

[fsn33874-bib-0032] Pathare, P. B. , Opara, U. L. , & Al‐Said, F. A. J. (2013). Colour measurement and analysis in fresh and processed foods: A review. Food and Bioprocess Technology, 6(1), 36–60. 10.1007/s11947-012-0867-9

[fsn33874-bib-0033] Rahman, A. A. (1986). Chemical and physical characteristics of pepper mash and hot pepper sauce. Dissertation, 183, 28–29.

[fsn33874-bib-0034] Ramalingam, S. , Bahuguna, A. , Lim, S. , Joe, A.‐R. , Lee, J.‐S. , Kim, S.‐Y. , & Kim, M. (2022). Physicochemical, microbial, and volatile compound characteristics of *Gochujang*, fermented red pepper paste, produced by traditional cottage industries. Foods, 11(3), 375. 35159525 10.3390/foods11030375 PMC8834593

[fsn33874-bib-0035] Sasikumar, J. M. , Erba, O. , & Egigu, M. C. (2020). In vitro antioxidant activity and polyphenolic content of commonly used spices from Ethiopia Heliyon in vitro antioxidant activity and polyphenolic content of commonly used spices from Ethiopia. Heliyon, 6, e05027. 10.1016/j.heliyon.2020.e05027 32995654 PMC7511827

[fsn33874-bib-0036] Sayin, K. , & Arslan, D. (2015). Antioxidant properties, ascorbic acid and Total carotenoid values of sweet and hot red pepper paste: A traditional food in turkish diet. World Academy of Science, Engineering and Technology International Journal of Nutrition and Food Engineering, 9(7), 834–837.

[fsn33874-bib-0037] Sharifi, A. , Niakousari, M. , Maskooki, A. , & Mortazavi, S. A. (2015). Effect of spray drying conditions on the physicochemical properties of barberry (Berberis vulgaris) extract powder. International Food Research Journal, 22(6), 2364–2370.

[fsn33874-bib-0038] Tekin, Z. H. , & Baslar, M. (2018). The effect of ultrasound‐assisted vacuum drying on the drying rate and quality of red peppers. Journal of Thermal Analysis and Calorimetry, 132(2), 1131–1143. 10.1007/s10973-018-6991-7

[fsn33874-bib-0039] Tigu, F. , Assefa, F. , Mehari, T. , & Ashenafi, M. (2016). Probiotic property of lactic acid bacteria from traditional fermented condiments: Datta and awaze. International Food Research Journal, 23(2), 770–776.

[fsn33874-bib-0040] Tsegaye, M. , Ephraim, E. , & Ashenafi, M. (2004). Behaviour of Escherichia coli O157:H7 during the fermentation of Datta and awaze, traditional Ethiopian fermented condiments, and during product storage at ambient and refrigeration temperatures. Food Microbiology, 21(6), 743–751. 10.1016/j.fm.2004.02.003

[fsn33874-bib-0041] Yang, H. J. , Lee, Y. S. , & Choi, I. S. (2018). Comparison of physicochemical properties and antioxidant activities of fermented soybean‐based red pepper paste, gochujang, prepared with five different red pepper (Capsicum annuum L.) varieties. Journal of Food Science and Technology, 55(2), 792–801. 10.1007/s13197-017-2992-y 29391645 PMC5785406

[fsn33874-bib-0042] Yo, B. (2001). Rheological properties of hot pepper‐soybean paste. Journal of Texture Studies, 32, 307–318.

[fsn33874-bib-0043] Zhuang, Y. , Chen, L. , Sun, L. , & Cao, J. (2012). Bioactive characteristics and antioxidant activities of nine peppers. Journal of Functional Foods, 4(1), 331–338. 10.1016/j.jff.2012.01.001

